# Herd Immunity and the Harm Principle: Too Narrow or Too Broad

**DOI:** 10.1093/phe/phag014

**Published:** 2026-07-13

**Authors:** Justin Bernstein

**Affiliations:** Corcoran Department of Philosophy, University of Virginia, 120 Cocke Hall, Charlottesville, Virginia, 22904-4780, USA

## Abstract

In this commentary, I will contend that one of Pierik and Verweij’s central arguments for vaccine mandates faces significant obstacles because of their attempt to apply Mill’s harm principle. More specifically, they argue that vaccine refusal is harmful because of its relationship to the collective good of herd immunity. I suggest that Pierik and Verweij oscillate between two interpretations of the harm principle: a narrow interpretation and a broad one. The narrow interpretation, however, does not justify mandates because it is implausible that one individual’s act of vaccine refusal makes a meaningful difference to whether herd immunity is achieved. The broad interpretation avoids this problem, but it risks abandoning the authors’ aspiration to advance a distinctively liberal argument. I conclude that those who wish to defend vaccine mandates by appealing to their role in achieving or maintaining herd immunity face a choice: they need to either (i) find argumentative resources within the liberal tradition that are more promising than the harm principle or (ii) develop defenses that are not liberal but indicate why this does not constitute a problem.

In *Inducing Immunity?*, Roland Pierik and Marcel Verweij (henceforth P&V) provide a timely, systematic and sophisticated defense of vaccine mandates. Several of P&V’s arguments appeal to their version of John Stuart Mill’s ‘harm principle’, which holds that restrictions of liberty are justified if they prevent harm to non-consenting third parties. Harm, in turn, involves setting back interests in a way that violates rights Pierik and Verweij, 2024: p. 59). P&V appeal to the harm principle both because it seems promising at justifying liberty-restricting measures like vaccine mandates, and because the principle enjoys widespread acceptance among liberals—indeed, it is ‘one of the cornerstones of the contemporary liberal-democratic tradition in legal and political philosophy,’ and ‘every democrat, whether egalitarian, libertarian, communitarian, or socialist, must [accept the harm principle]’ (p. 59).

This commentary will focus on P&V’s arguments for justifying vaccine mandates given the connection between vaccine refusal, herd immunity, and the harm principle. P&V intend these arguments to apply both when vaccination rates are meaningfully below (pp. 66–67) and above (pp. 67–71) the threshold for herd immunity. In what follows, I will suggest that P&V oscillate between two arguments that rest on distinct interpretations of the harm principle identified by Mill scholars.^1^ I will contend that each interpretation faces significant obstacles given P&V’s argumentative goals. P&V’s argument that invokes the ‘Narrow Interpretation’ of the harm principle fails because individual acts of vaccine refusal do not make enough of a difference to herd immunity to set back interests. While P&V’s argument invoking the ‘Broad Interpretation’ of the harm principle avoids this problem, it risks becoming less distinctly liberal—and thus stands in tension with one of P&V’s main stated aims for the book.

## The Narrow Interpretation Is Too Narrow

The Narrow Interpretation of the harm principle applies only to restricting individuals’ liberty to prevent them from performing harmful *actions*. Harmful actions are those that set back interests and violate individual rights or pose a sufficiently high risk of doing so. For example, laws forbidding drunk driving are justified by preventing the direct risk of harm to other drivers. But the Narrow Interpretation would not countenance broader restrictions on alcohol consumption on the grounds that doing so would increase worker productivity or reduce alcohol-related diseases—and associated gains for the public interest—or prevent indirect harms that arise from setting a bad example for other adults.

P&V join others by contending that the Narrow Interpretation may justify mandates in contexts where vaccination rates are sufficiently low—assuming that one unvaccinated individual imposes sufficiently high risk on third parties.^2^ But can vaccine refusal be considered a harmful action in virtue of its effects on herd immunity? P&V contend that it can. To secure this conclusion, they first lean on Mill’s remarks that invoke the harm principle ‘as a justification for compelling members of society to contribute to collective projects that benefit all’ (p. 63). P&V then make a similar argument that focuses on *undermining* the collective effort to produce collective goods—indeed, they go as far as to accord this argument pride of place: ‘Vaccine refusal can be considered harmful to others in various ways…*Most important in our analysis is that it undermines the collective endeavour to protecting* [sic] *society via herd immunity*’ (p. 196, emphasis added).

Suppose we apply the Narrow Interpretation of the harm principle to these claims about vaccine refusal, harm, collective benefits, undermining, and herd immunity. We then have the following argument:

### The Narrow Argument

P1. The Narrow Interpretation: If an act harms others, the government may justifiably use liberty-restricting measures to prevent that action.

P2. If an act constitutes (a) a failure to contribute to a collective effort to produce or maintain an indispensable, collectively achieved good, then that action harms others. (pp. 63, 65)^3^

P3. If an act (b) undermines a collective effort to achieve or maintain an indispensable, collectively achieved good, then that action harms others. (pp. 67–71)

P4. Vaccine refusal (on behalf of oneself or one’s children) constitutes an instance of (a) or (b) because the acts fail to contribute to or undermine herd immunity. (pp. 63–71)

C. So, governments may use liberty-restricting measures to ensure that individuals get themselves or their children vaccinated.

While this argument is valid, we should reject P2 and P4.

Beginning with P2, one individual’s failure to contribute to a sufficiently large collective effort does *not* make a meaningful difference to the success or failure of the collective effort.^4^ Since one individual’s action does not make a meaningful difference, their (in)action does not set back others’ interests. And an action is harmful only if it sets back interests. So, we should reject the claim that acts of type (a) constitute harms. Of course, we can still criticize non-contributors on various grounds—e.g. that they violate prohibitions on free riding.^5^ However, the question for The  Narrow  Argument is whether we should understand individual acts of vaccine refusal as *harms* in virtue of their connection to herd immunity. So, we should reject P2.

P3 provides a different route to the same conclusion. More precisely, even though we have seen above that we should not accept P2 because acts of type (a) do not constitute harms, if acts of type (b) constitute harms, and vaccine refusal is an act of type (b), then P3 and P4 could still secure P&V’s conclusion. Moreover, P3 seems more plausible than P2. Imagine a group is constructing a communal well, but every evening, when they stop work, a malevolent individual uses a sledgehammer to make a meaningful difference to its structural soundness. Presumably, this sets back the interests of community members in a way that violates their rights—a case of undermining a collective endeavor that also constitutes a harm. However, the case of vaccine refusal is not like this, so, we should reject P4. One act of vaccine refusal does *not* make a meaningful difference to the success of the collective endeavor to establish herd immunity; the endeavor will fail only if a sufficiently high percentage of the (large) population refuses vaccination. So, a particular instance of vaccine refusal does not appear to count as setting back anyone’s interests in virtue of its effects on the collective effort to produce the collective benefit of herd immunity. Even if vaccine refusal is an instance of wrongful ‘undermining’ in some sense, the act is not wrongful in virtue of setting back interests in a way that violates rights, and so it is not a harmful action in the sense needed for The  Narrow  Argument to succeed.

## Defending the Narrow Interpretation?

My objections to P2 and P4 have each turned on the fact that the relevant (in)actions do not make a meaningful difference to producing or maintaining the collectively achieved good of herd immunity. Since the relevant (in)actions do not make a meaningful difference, they do not count as setting back some individual’s interests in a way that violates their rights and thus are not harms.^6^ The authors acknowledge this objection, but they defend The  Narrow  Argument against it:

If one individual’s actions do not seem to make much of a difference to the harm that might occur—but would occur only if many acted like them—how can their choice be considered a case of harm to others? For several reasons, we believe…skepticism about collective harm is misplaced. (pp. 68–69)

One such reason builds on the observation that ‘even if hesitant and actively refusing parents are still a minority, their number is large enough to obstruct establishment of such a high coverage’ (p. 69). But no one denies that sufficiently widespread vaccine refusal can undermine herd immunity. The question for The  Narrow  Argument is whether each *individual* act of refusal makes enough of a difference to the collectively achieved good to count as setting back other interests. So, appealing to the group’s harmful effects does not show that an individual act of vaccine refusal constitutes a harm.

In a second response, P&V claim that when you fail to contribute to herd immunity,

…you create a weakness in collective protection that cannot be compensated for by others…in this way, each and every failure to collaborate generates a weakness in the protection of public health and, as such, undermines the collective endeavor at large. (p. 70)

But whether someone else can ‘compensate’ for your non-contribution appears to be irrelevant to whether an individual act constitutes a harm. For example, consider Derek Parfit’s case of the harm*less* torturers, where 1000 torturers each contribute a negligible amount of electric current that, collectively, causes an agonizing shock to 1000 victims.^7^ As a variation of that case, imagine that each button that sends electric current to the victims requires the thumbprint of a particular torturer. In this scenario, each of the 1000 torturers is uniquely capable of contributing a (negligible) portion of electric current in a way that ‘could not be compensated for by others.’ Given that each torturer makes a negligible causal contribution to the victims’ pain, however, the case still appears to be a case of harm*less* torturers. Returning to vaccination, the worry for P&V is that an individual’s contribution to herd immunity is not causally significant enough to count as setting back interests—even if others cannot compensate for one individual’s (negligible) contribution to herd immunity.

In a third response, P&V contend that vaccine refusal undermines the collective endeavor to establish herd immunity by setting a bad example for others:

…if [parents susceptible to vaccination concerns] see others just ‘opting out’ with no clear consequences, this will make them wonder why they should comply; it facilitates seeing opting out as a reasonable and legitimate option and thus nudges them to forgo immunization as well. In these ways, individual vaccination refusals constitute a collective harm…(p. 70.)

However, P&V’s argument encounters a version of the worry that the argument aims to address. Namely, for most individuals, an act of vaccine refusal is not influential enough on others to make a meaningful difference to whether the collective endeavor of achieving herd immunity succeeds or fails. Perhaps more importantly, this response claims that setting a bad example constitutes a harm and thus licenses liberty restriction. But this abandons the Narrow Interpretation of the harm principle, which contends that liberty restrictions are justified to prevent acts that *directly* set back someone else’s interests. Instead, this reply rests on an indirect understanding of harm.^8^

## The Broad Interpretation Is Too Broad

A natural response at this point would be to embrace the relevant indirect understanding of harm. And while I remain convinced that P&V intend to make The  Narrow  Argument, they make other remarks and cite others in a way that suggests that they also intend to advance a distinct argument that embraces this indirect understanding of harm.^9^

The Broad Interpretation of the harm principle holds that governments are permitted to restrict liberty to prevent *harmful states of affairs*, not just individually harmful *actions*. On this reading, even if no individual act of vaccine refusal constitutes a harm in virtue of failing to contribute to/undermining herd immunity, the aggregation of acts of vaccine refusal causes harm because this will result in the loss of herd immunity, which will, in turn, put various individuals at risk of infection. Thus, on the Broad Interpretation, the government may restrict liberty by implementing mandates. So, if the Broad Interpretation of the Harm Principle is viable, then P&V should be interpreted as follows:

### The Broad Argument

P1. The Broad Interpretation: If restricting the liberty to perform some action prevents harm from occurring, then there is a (pro tanto) justification for such liberty-restricting measures.

P2. Restricting the liberty to refuse vaccination for oneself or one’s children prevents harm from occurring.

C. So, there is a (pro tanto) justification for the government to restrict vaccine refusal.

The  Broad  Argument avoids the objections I raised for The  Narrow  Argument. Yet P1 risks abandoning the *liberal* character of P&V’s defense of mandates.

This risk arises because the Broad Interpretation enlarges the range of legitimate restrictions of liberty in two respects. The first respect concerns the *object* of evaluation. While the Narrow Interpretation merely assesses whether particular *actions* are harmful, the Broad Interpretation also evaluates whether *states of affairs* that result from aggregations of acts constitute harms. Second, and relatedly, the Broad Interpretation enlarges the *types* of acts that may be legitimately restricted. Once the warrant for coercion includes preventing harmful states of affairs, there is no reason to limit coercion to *directly* harmful actions. After all, various kinds of indirectly harmful act-types—e.g. negatively influencing others, to use an example from P&V’s discussion—can, in the aggregate, result in harmful states of affairs, even if individually such acts do not directly harm anyone else.

The illiberal potential of these two enlargements might already be apparent to readers. Nonetheless, to further illustrate this illiberal potential, I will draw on historical examples in which proponents of a policy attempted to justify it by appealing to something like the Broad Interpretation. To be clear, in invoking these examples do not mean to suggest that the relevant cases are morally comparable to vaccine refusal; instead, I aim to highlight how readily the Broad Interpretation lends itself to illiberal arguments.

To begin, as Bernard Harcourt and Steven Smith point out, conservatives have appealed to the harm principle to justify restricting same-sex relationships on the grounds that such relationships cause social decay, whereas radical feminists have appealed to the harm principle to justify censorship of heterosexual pornography, contending that the consumption of such pornography (taken collectively and indirectly) harms women.^10^ Proponents of motorcycle helmet mandates in the United States quickly invoked harms to third parties that would result from the unnecessary expenditures on medical care that accompany accidents of helmetless riders.^11^ In each of these cases, the Broad Interpretation of the harm principle is invoked to prevent the loss of some collective good—traditional family values, appropriately respectful societal attitudes towards women, communally available medical resources—which, if lost, could result in harm to some third parties. Even if one finds one or more of these arguments compelling, they seem paradigmatically *il*liberal since they constrain consenting adults from exercising core basic liberties—e.g. freedom of association, freedom of the person—for the sake of preventing the loss of some collective good and the accompanying relevant indirect threats of harm.^12^

At this juncture, many readers would (correctly) observe that achieving or maintaining herd immunity differs importantly from these examples. For example, presumably, many readers share my deep skepticism about the conservative argument concerning the effects of tolerating same-sex relationships, or they might point out that achieving herd immunity involves far fewer enforcement obstacles or unintended negative consequences than censoring heterosexual pornography. But I appeal to these examples for two reasons.

First, I aim to highlight the argumentative form these examples share with P&V’s defense of mandates. If the enlarged scope of the Broad Interpretation so readily lends itself to illiberal conclusions in other cases of conflict between collectively achieved goods and individual liberty, we should be skeptical that the Broad Interpretation constitutes a liberal justification in the case of vaccine mandates. And these examples *do* suggest that the enlarged scope of the Broad Interpretation does just this.

Second, even if one balks at this skepticism, these examples highlight how invoking the Broad Interpretation incentivizes advocates of various perfectionist or paternalist goals to redescribe their policies in terms of preventing collective harm. Insofar as so many other illiberal arguments get framed in the same terms, we should worry that framing vaccine mandates in terms of the relevant collective harm will not be received as a genuinely liberal argument, or that its rhetorical power will be diminished because of the proliferation of structurally similar arguments for illiberal policies.

Of course, P&V need not proffer a liberal defense of vaccine mandates. However, as noted in the introduction, this is a core aim of the book—in part because all liberals must accept the harm principle (p. 59). This claim about universal acceptance seems plausible enough for the Narrow Interpretation, but the Narrow Interpretation cannot do the relevant argumentative work that P&V need it to do. The Broad Interpretation can do the relevant argumentative work, but it’s implausible that it is accepted by a broad variety of liberals.^13^ Thus, those who wish to defend vaccine mandates by appealing to their role in achieving or maintaining herd immunity face a choice: they need to either (1) find argumentative resources within the liberal tradition that are more promising than the harm principle, or (2) develop defenses that are not liberal and indicate why this does not constitute a problem. I suspect (1) offers the more promising route, and in my own work with Mark Navin, we argue that when justifying mandates by appealing to their role in achieving or maintaining herd immunity, we should appeal to liberal theories of justice rather than the harm principle.^14^ However, further elaborating on this claim will have to wait until a later date.

## Notes

1 See, e.g. Gray (1996: 58–59 and Brink (2022).

2 See Flanigan (2014: 63). P&V acknowledge that when vaccination rates are sufficiently high, it's less clear that an individual act of vaccine refusal imposes sufficient risk to trigger the harm principle. It is worth noting, however, that some are skeptical that this argument succeeds—even in the context where vaccination rates are quite low. See esp. Steinhoff (2024: 1–3) and Giubilini (2020).

3 It is worth noting that others express skepticism that Mill’s harm principle applies to cases of failing to contribute. For instance, David Brink expresses misgivings about treating failures to contribute to public goods as harms: “the impact of individual failures to contribute to public goods is too small and spread too widely to constitute the breach of ‘a distinct and assignable obligation to any other person or persons’” (Brink, 2022).

4 c.f. Nefsky (2017).

5 Although P&V expressly reject free riding arguments—see pp. 72–81. For a nearby argument that appeals to requirements of justice without appealing to free riding, see esp. Bernstein and Navin (2023).

6 For an overview of literature on this problem, see Nefsky (2019).

7 (Parfit, 1984). For an application to the context of vaccine mandates, see Giubilini (2020).

8 ‌We have also departed significantly departed from Mill's own understanding of the harm principle, which rejects setting a bad example for the broader community as sufficient grounds for liberty-restriction See esp. Mill (2006: Chapter IV).

9 See the references to Elizabeth Cripps and David Lyons (pp. 68–70).

10 See esp. Harcourt (1999) and Smith (2004).

11
Jones and Bayer (2007).

12 For similar concerns about extending the harm principle, see e.g. Feinberg (1987: 222).

13 Libertarians do not think that refusal to contribute to collective goods can constitute rights violations. For classic statements of this view, see Nozick (1974) and Simmons (1979). For an argument along these lines in the context of vaccine refusal, see Bernstein (2017).

14 See esp. Bernstein and Navin (2023).
